# Reporting standards for economic evaluations of artificial intelligence interventions: a CHEERS extension

**DOI:** 10.1017/S0266462324004756

**Published:** 2024-11-29

**Authors:** Gurdeep S. Sagoo, Marina Richardson

**Affiliations:** International Journal of Technology Assessment in Health Care (IJTAHC), Toronto, ON, Canada

The International Society for Pharmacoeconomics and Outcomes Research (ISPOR) Health Economic Evaluation Publication Guidelines Good Reporting Practices Task Force published the first Consolidated Health Economic Evaluation Reporting Standards (CHEERS) statement (1) in 2013 to provide a set of recommendations to optimize the reporting of health economic evaluations. The statement provides examples and explanations for the recommendations in an easy-to-complete twenty-four-item checklist. It has subsequently been highly cited and adopted by many international journals as a prerequisite to article submission and, as such, is familiar to anyone conducting and submitting an economic evaluation for publication. In 2022, the reporting guidance was updated (2) and replaced the original version with a twenty-eight-item checklist to enhance the original statement by addressing calls to recognize new developments in the field including the growth of patient and public involvement in research. An extension to the CHEERS 2022 statement has recently been published to ensure that economic evaluations of artificial intelligence (AI)-based health interventions are reported in a sufficiently transparent manner (3).

The publication of CHEERS-AI is timely, given the increasing focus on the development and use of AI in the healthcare system. Syntheses of economic evaluations of AI-based interventions are common (4–7) and have found that published economic evaluations of AI-based interventions are generally of moderate quality when it comes to following reporting guidelines (6), including, specifically, adequately reporting the details of the AI nature of the intervention (5). The development of this CHEERS-AI extension is prudent to ensure that such studies are reported as transparently as possible. The CHEERS-AI extension is built on the existing 2022 update but provides specific guidance relevant to AI-based interventions. It was developed through several steps: 1) convening of an expert steering group, 2) creation of a long list of possible reporting items, 3) refining the long list to take forward to a consensus development phase, 4) a three-round consensus Delphi exercise, 5) consensus meeting, 6) engagement with a patient expert group, 7) piloting of the agreed reporting items, and 8) final ratification of the CHEERS-AI checklist items. This process led to a thirty-eight-item checklist consisting of the twenty eight items included within the 2022 update plus ten new AI-specific reporting items. Furthermore, eight of the existing items now have additional guidance to draw out the relevance to AI more clearly. If implemented by editors, peer reviewers, and authors, the checklist is anticipated to lead to the inclusion of details about the AI technology that may not have been included in the reporting of an economic evaluation before the development of the guidance.

The AI extension calls for specific delineation of aspects of AI, such as how the intervention affects clinical care (e.g., diagnosing, treating, or informing clinical management); describing the details of the AI component, including how the effect of the AI is measured, how AI learning occurs over time, and how the AI component was developed and validated; reporting the results of analyses of uncertainty; and discussing the necessary requirements for implementation.

We commend the authors, and the authors of the core CHEERS checklist, for continuing to promote the publication of economic evaluations that are clearly, consistently, and transparently reported. We encourage authors to consider both CHEERS and CHEERS-AI in their submissions of economic evaluations to our journal.
